# Recent Advances in Incretin-Based Pharmacotherapies for the Treatment of Obesity and Diabetes

**DOI:** 10.3389/fendo.2022.838410

**Published:** 2022-03-01

**Authors:** Qiming Tan, Seun E. Akindehin, Camila E. Orsso, Richelle C. Waldner, Richard D. DiMarchi, Timo D. Müller, Andrea M. Haqq

**Affiliations:** ^1^ Department of Pediatrics, University of Alberta, Edmonton, AB, Canada; ^2^ Institute for Diabetes and Obesity, Helmholtz Diabetes Center at Helmholtz Zentrum München, Germany and German Center for Diabetes Research (DZD), Munich, Germany; ^3^ Department of Agricultural Food & Nutritional Science, University of Alberta, Edmonton, AB, Canada; ^4^ Department of Chemistry, Indiana University, Bloomington, IN, United States

**Keywords:** incretin, GLP-1, GIP, obesity, diabetes, drug

## Abstract

The incretin hormone glucagon-like peptide-1 (GLP-1) has received enormous attention during the past three decades as a therapeutic target for the treatment of obesity and type 2 diabetes. Continuous improvement of the pharmacokinetic profile of GLP-1R agonists, starting from native hormone with a half-life of ~2–3 min to the development of twice daily, daily and even once-weekly drugs highlight the pharmaceutical evolution of GLP-1-based medicines. In contrast to GLP-1, the incretin hormone glucose-dependent insulinotropic polypeptide (GIP) received little attention as a pharmacological target, because of conflicting observations that argue activation or inhibition of the GIP receptor (GIPR) provides beneficial effects on systemic metabolism. Interest in GIPR agonism for the treatment of obesity and diabetes was recently propelled by the clinical success of unimolecular dual-agonists targeting the receptors for GIP and GLP-1, with reported significantly improved body weight and glucose control in patients with obesity and type II diabetes. Here we review the biology and pharmacology of GLP-1 and GIP and discuss recent advances in incretin-based pharmacotherapies.

## 1 Introduction

**Figure 1:**
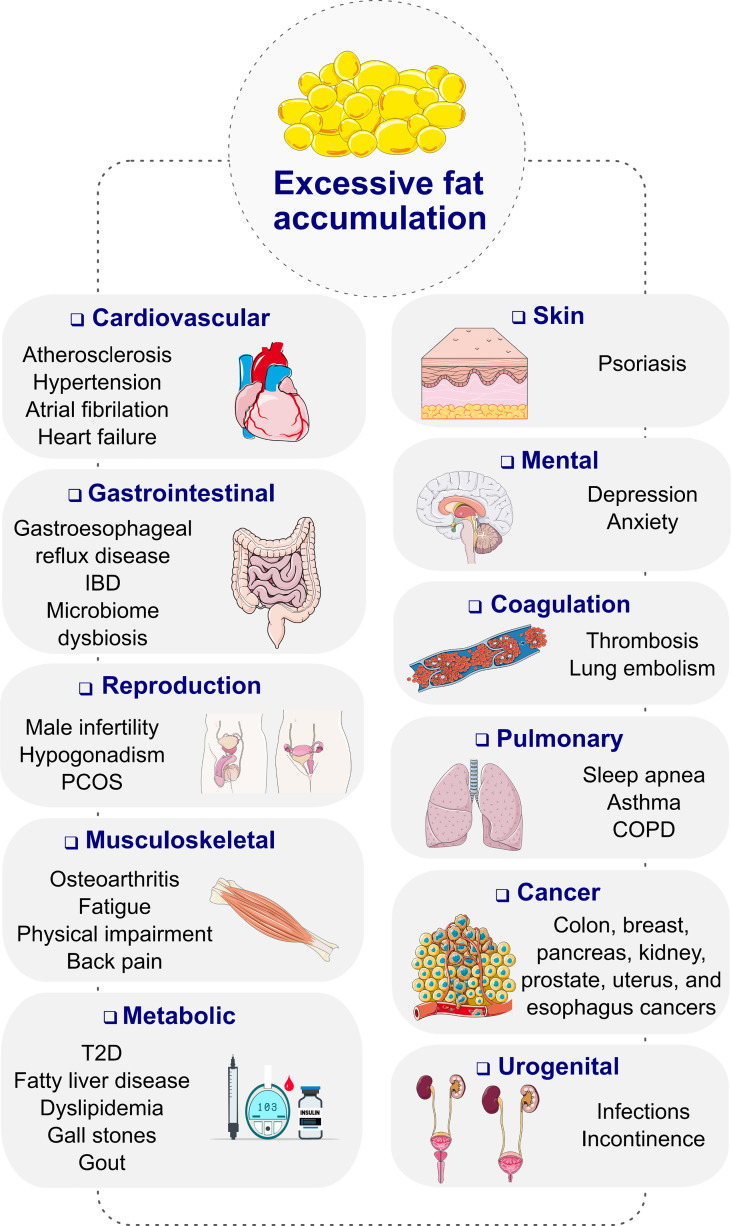
Complications of obesity. COPD, chronic obstructive pulmonary disease; IBD, inflammatory bowel disease; PCOS, polycystic ovary syndrome; T2D, type 2 diabetes. Images retrieved from smart.servier.com.

Obesity, diagnosed as a body mass index (BMI) ≥ 30 kg/m^2^, is a progressive, chronic disease that has grown to pandemic prevalence over the past decades (1). Obesity substantially increases the risk of type-2 diabetes (T2D), cardiometabolic diseases, osteoarthritis, neurological and mental disorders as well as several forms of cancer, resulting in premature disability and demise (**Figure 1**) (2, 3). Depending on the severity of the disease and the age at diagnosis, long-term health complications may last a lifetime and worsen the therapeutic outcome for multiple associated chronic diseases (2). Unsurprisingly, obesity leads to excess medical costs and imposes a large economic burden on individuals, families, health care systems, and societies (2, 4).

While traditionally recognized primarily as a disease of the elderly, T2D is currently one of the most frequently diagnosed preventable chronic diseases in middle age, as well as children and adolescents (5, 6). Excess body fat along with age constitute the two most important risk factors for the premature development of T2D. Some studies report that >80% of youth and ~50% of adults with T2D are overweight or obese at point of diagnosis (6, 7). Early onset T2D relative to late-onset disease is associated with a more rapid deterioration of β-cell function, emphasizing the importance for early diagnosis and treatment initiation (8). Obesity-related mechanisms that are potentially linked to the severity of the disease include adipocyte lipid spillover, ectopic fat accumulation and tissue inflammation (9). Sizeable weight loss not only improves glucometabolic health, it may also reduce the risk for obesity-linked co-morbidities, increase life expectancy, and improve quality of life (10–12). Therapies aiming to decrease body weight are consequently a valuable strategy to delay the onset and decrease the risk of T2D, as well as managing established disease (13).

Lifestyle modifications, such as balanced nutrition, calorie restriction and physical exercise, remain the cornerstone of any weight loss intervention. However, lifestyle changes alone are insufficiently efficacious and sustainable as a stand-alone therapy, possibly because physiological adaptations conspire to promote weight regain following diet-induced weight loss (14). Genetic and environmental factors may further undermined treatment efficacy (15). Polygenetic gene variants, each accounting for only a small difference in body weight, may sum up to sizably affect body mass and may hinder susceptibility of an individual to respond to a weight loss intervention (16, 17). There are also less frequent variants with larger effects leading to early onset of severe obesity in humans. Several syndromic and monogenic disorders of obesity that have been identified include Prader–Willi syndrome, Bardet–Biedl syndrome, and loss-of-function mutations in the genes encoding for pro-opiomelanocortin (POMC), leptin, leptin receptor (LEPR) or the melanocortin-4 receptor (MC4R) (17).

Pharmacotherapy as an adjunct to lifestyle adjustments is often used to enhance weight loss efficacy (18). However, a key obstacle in the development of anti-obesity medication is that rodent studies proved largely incapable to predict cardiovascular safety in humans (13, 19, 20). Also, the heterogeneity of patient cohorts, with many individuals being of advanced age and at high risk for development of cardiovascular diseases (CVD), represents an obstacle that is not easy to address with pharmacotherapy (19). Consistent with this, a series of previously employed anti-obesity medications were withdrawn soon after approval due to unforeseen adverse effects on the cardiovascular system (19–21). Furthermore, when given at tolerable doses, pharmacotherapy rarely decreases body weight >10%. Notable exceptions are semaglutide 2.4 mg (Wegovy^®^ Novo Nordisk, Copenhagen, Denmark), a long-acting agonist at the glucagon-like peptide-1 receptor (GLP-1R) (22), and the experimental drug candidate tirzepatide, a dual-agonist at the receptors for GLP-1 and the glucose-dependent insulinotropic polypeptide (GIP) (23). Each of these peptides decrease body weight with a favorable safety profile in the majority of patients by >10% (24–28). While the clinical success of these drugs sets the stage for a new era in anti-obesity medication, there remains considerable controversy as to how GIP regulates metabolism and whether GIP receptor agonism or antagonism is a preferred treatment for obesity and T2D. In this manuscript, we provide an overview of the mechanistic biology and *in vivo* pharmacology of GLP-1 and GIP. We summarize recent clinical results with molecules that target each receptor and discuss recurrent questions related to their mode-of-action.

## 2 Glucagon-Like Peptide-1 (GLP-1)

### 2.1 The Physiology of GLP-1

GLP-1 is encoded by proglucagon, a 158 amino acid precursor protein, that is predominantly expressed in the gut, pancreas, and distinct neuronal populations of the hindbrain (29). In the brain and the intestine, proglucagon is cleaved by the action of the prohormone convertase 1/3 (PC1/3) into GLP-1, GLP-2, glicentin, glicentin-related polypeptide (GRPP), and oxyntomodulin (OXM) (29–31). In the pancreatic α-cells, proglucagon is cleaved by PC2 into glucagon, GRPP and the major proglucagon fragment (MPGF). In the intestine, GLP-1 is secreted from enteroendocrine L-cells located in the gut epithelium. The density of the L-cells is low in the duodenum and jejunum and it is high in the ileum and colon (29). Nutrients stimulating the secretion of GLP-1 in the intestine include monosaccharides such as glucose, galactose, fructose (32–34), fatty acids (35, 36), as well as proteins (37) and amino acids, particularly glutamine and glycine (38, 39). The relevance of endocrine factors to promote GLP-1 secretion seem to vary across species and may include acetylcholine, insulin, ghrelin, GIP, and gastrin-releasing peptide (29). Circulating levels of total GLP-1 are low during fasting (~5 pmol/l) and rapidly rise up to 40 pmol/l shortly after a meal (40). Consistent with the ability of GLP-1 to accelerate glucose-stimulation of insulin secretion (GSIS), the meal-induced rise in plasma GLP-1 is paralleled by enhanced insulin immunoreactivity (40).

GLP-1 promotes its biological action through binding to the GLP-1 receptor (GLP-1R), a 7 transmembrane G protein-coupled receptor of the class B family (41). GLP-1R signals primarily *via* the Gαs pathway, and hence accelerates intracellular levels of cAMP (42). GLP-1R can also recruit, and induce signaling, *via* the Gαq and β-arrestin pathways and knockdown of β-arrestin-1 in rat insulinoma (INS1) cells decreases the ability of GLP-1 to promote GSIS (43). Immunohistochemical studies in tissues from humans and non-human primates show widespread distribution of GLP-1R in the brain and in the periphery (44). These data largely align with studies in rodents in which the abundance of the GLP-1R transcript was assessed using mice that express green fluorescent protein (GFP) under control of the GLP-1R promoter (45). Consistent with the ability of GLP-1R agonists to decrease homeostatic and hedonic food intake (29, 29), expression GLP-1R is found in the rodent hypothalamus (ARC, VMH, DMH, PVH, LH), hindbrain (AP, NTS, ventrolateral medulla) and telencephalon (amygdala, olfactory bulb, preoptic area, nucleus accumbens) (45, 46). In the pancreas, GLP-1R is solidly expressed in the β- and δ-cells but is only found in a small portion of α-cells (45). No expression of GLP-1R is found in the liver and the thyroid gland (44).

**Figure 2:**
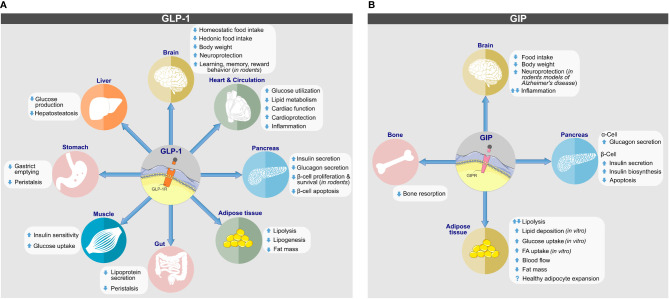
Biological actions of **(A)** GLP-1 and **(B)** GIP on target tissues. Direct and indirect effects are depicted. Images retrieved from smart.servier.com.

Albeit best known for its glycemic effects, GLP-1 is a pleiotropic hormone with a series of metabolic effects beyond the regulation of glucose metabolism (**Figure 2A**). Apart from its ability to act on the pancreas to enhance GSIS and to inhibit the secretion of glucagon, GLP-1 decreases body weight by decreasing homeostatic and hedonic food intake (29). GLP-1R agonism further inhibits gastric emptying (47); has cardio- and neuroprotective effects (48); lowers inflammation and apoptosis (49–53); stimulates β-cell proliferation in rodents (54); and exerts positive effects on learning, memory, and reward behavior (55, 56). Endogenous GLP-1 is mainly produced as GLP-1(7-36NH_2_), with a low proportion produced as GLP-1(7-37) and an even a lower portion as GLP-1(1-37) or GLP-1(1-36NH_2_) (29, 57). Native GLP-1 has a half-life of just ~2–3 min (58–60), which results mainly from rapid *in vivo* proteolysis by the dipeptidylpeptidase-4 (DPP-4) and fast renal elimination. DPP-4 cleaves GLP-1(7-36NH_2_) and GLP-1(7-37) at the second N-terminal amino acid (Ala8) position, leading to metabolically metabolites GLP-1(9-36NH_2_) and GLP-1(9-37) of much reduced potency (61). Despite species-related differences, GLP-1 is also subject to degradation by the neutral endopeptidase (NEP) 24.11, which cleaves GLP-1 at its central and C-terminal positions Asp15, Ser18, Tyr19, Glu27, Phe28 and Trp31 (61, 62). The relevance of NEP 24.11 to cleave GLP-1 varies among species and while it contributes substantially to GLP-1 degradation in mice and pigs (63, 64), it’s relevance in humans has long been questioned. Nonetheless, more recent data show that NEP 24.11 also plays a physiological relevant role for degradation of GLP-1 in humans (65).

Consequential due to its short half-life, native GLP-1 has only limited pharmacological potential and only ~10–15% of active GLP-1 is estimated to reach the general circulation (59, 66–68). Nonetheless, emphasizing its therapeutic implication, 6-wk continuous infusion of GLP-1(7-36NH_2_) at a rate of 4.8 pmol^-1^ kg^-1^ min^-1^ improved glycemic control and insulin sensitivity in patients with T2D (69). Despite the lack of singular attribution to enhanced GLP-1 action, similar results have been demonstrated following administration of a DPP-4 inhibitor (70–73).

Notably, as comprehensively reviewed elsewhere (20, 29, 74), GLP-1 improves glycemic control *via* several complementary mechanisms. In the pancreas, GLP-1 directly acts on the β-cells to promote GSIS *via* the action of PKA and Epac2 (29). Both pathways are equally important for the insulinotropic effect of GLP-1 and ensure that GLP-1 primarily enhances insulin secretion under conditions of hyperglycemia (29). Other than stimulating the secretion of insulin in a glucose-dependent manner, GLP-1 also promotes the production of insulin *via* activation of Pdx1, which binds to the insulin promoter and activates its expression (29). GLP-1 also lowers blood glucose by inhibiting the release of glucagon and thus inhibiting hepatic glucose production (29). Clamp studies in patients with T2D indicate that the insulinotropic and glucagonostatic effects of GLP-1 contribute equally to decreasing blood glucose (75). While GLP-1 directly acts on the β-cells to stimulate insulin secretion, GLP-1 inhibition of glucagon secretion seems to be indirectly triggered *via* paracrine effects in the islets. Accordingly, GLP-1 stimulates the secretion of insulin, zinc, GABA, amylin, and somatostatin, all of which inhibit glucagon secretion (30). Supporting this notion of a paracrine effect is that the GLP-1 receptor is only expressed in a small subset of α-cells (45). However, GLP-1 does not only decrease blood glucose *via* its direct effects on the islets, it also inhibits gastric emptying and thereby slows glucose entry into the circulation (47, 76–78). Emphasizing the importance of GLP-1-mediated inhibition on gastric emptying for the regulation of blood glucose, antagonizing GLP-1’s effect on gastric emptying by co-infusion of GLP-1 with erythromycin during a liquid meal diminished GLP-1’s ability to decrease post-prandial hyperglycemia in patients with T2D (79).

In summary, GLP-1 improves fasting blood glucose through its direct action on the pancreatic islets and decreases postprandial hyperglycemia through inhibition of gastric emptying, and thus reduced glucose entry into circulation (74). These differences in glucose regulation translate into important pharmacological differences. Short-acting GLP-1 mimetics (e.g. exenatide BID, lixisenatide) are self-administered prior to a meal and, in conjunction with their short half-life of 2–3 h, display substantial fluctuations in circulation, with highest levels during the prandial and early post-prandial state, and lowest levels in the fasting periods between meals. Due to their relatively high plasma levels at the time of meal intake, the short-acting GLP-1 mimetics relative to the long-acting GLP-1R agonists display a higher tendency to affect GI motility and to decrease post-prandial hyperglycemia (74). On the contrary, long-acting GLP-1R agonists are less prone to affect GI motility and primarily decrease fasting blood glucose levels *via* direct action in the pancreas (74). With stable plasma concentrations and lower efficacy on GI motility, the long-acting GLP-1 mimetics are less prone to incur adverse gastrointestinal side effects (80) and generally have a higher potential to decrease body weight (74).

### 2.2 Pharmacological Advances in GLP-1R Agonism

**Figure 3:**
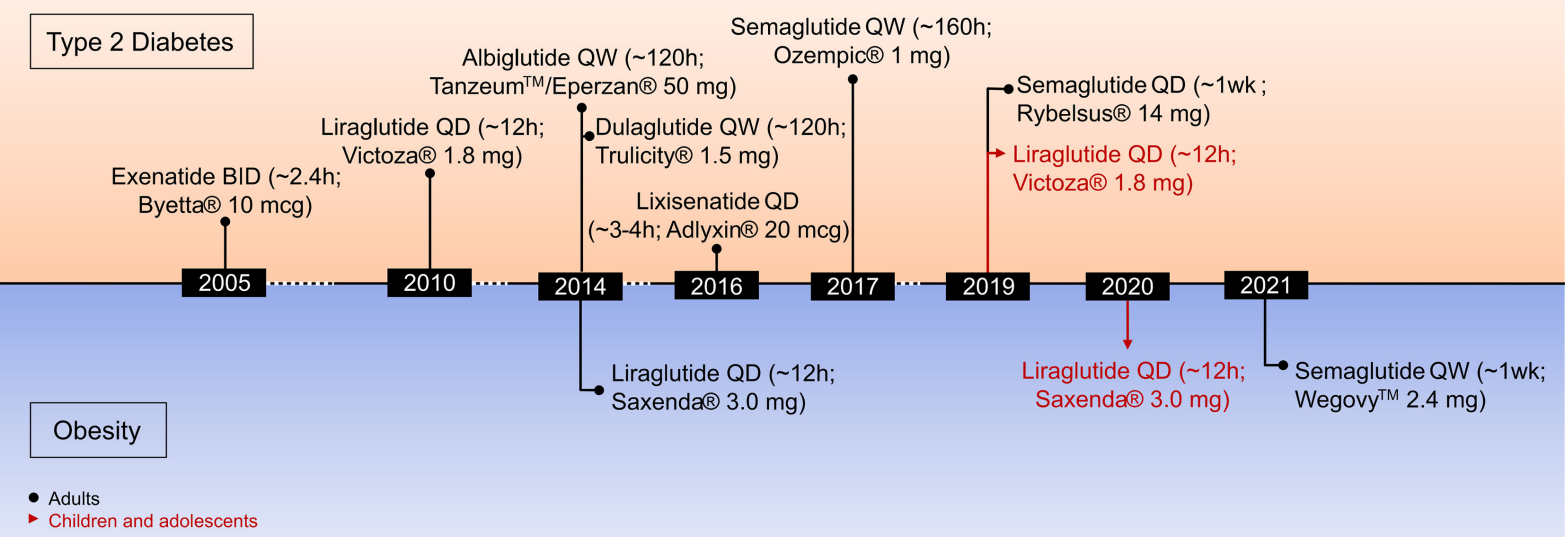
Timeline of drug approvals by the U.S. Food and Drug Administration.

A variety of structurally and chemically refined GLP-1R analogs have received extensive attention and have been implemented in clinical use for the treatment of T2D and obesity (**Figure 3**) (29). Clinical success in the treatment of obesity has been established for liraglutide 3 mg (Saxenda^®^, Novo Nordisk, Denmark) (81) and more recently for semaglutide 2.4 mg (Wegovy^®^, Novo Nordisk, Denmark) (13, 28). Also, molecules with simultaneous activity at the receptor for GIP (82–85) or glucagon (86, 87) have shown promising results for this application. The continuous improvement of the pharmacokinetic profile of GLP-1R agonism, starting from a native hormone with a half-life of ~2–3 min to the development of twice daily (exenatide BID), daily (liraglutide, lixisenatide) and even weekly (exenatide ER, albiglutide, dulaglutide, semaglutide) formulations, highlight the pharmaceutical advancement in this arena. Additionally, the recent development of an orally available preparation of semaglutide (Rybelsus^®^, Novo Nordisk), and the recruitment of GLP-1 into unimolecular pharmacology with GIP, glucagon (and others) (20, 29, 88) exemplifies how GLP-1-based drug development and innovation advanced over recent years. It emphasizes how structural refinements and biochemical modifications can extend the application of GLP-1R agonists from the treatment of T2D to obesity (13).

### 2.3 Methods to Extend GLP-1’s Half-Life

**Figure 4:**
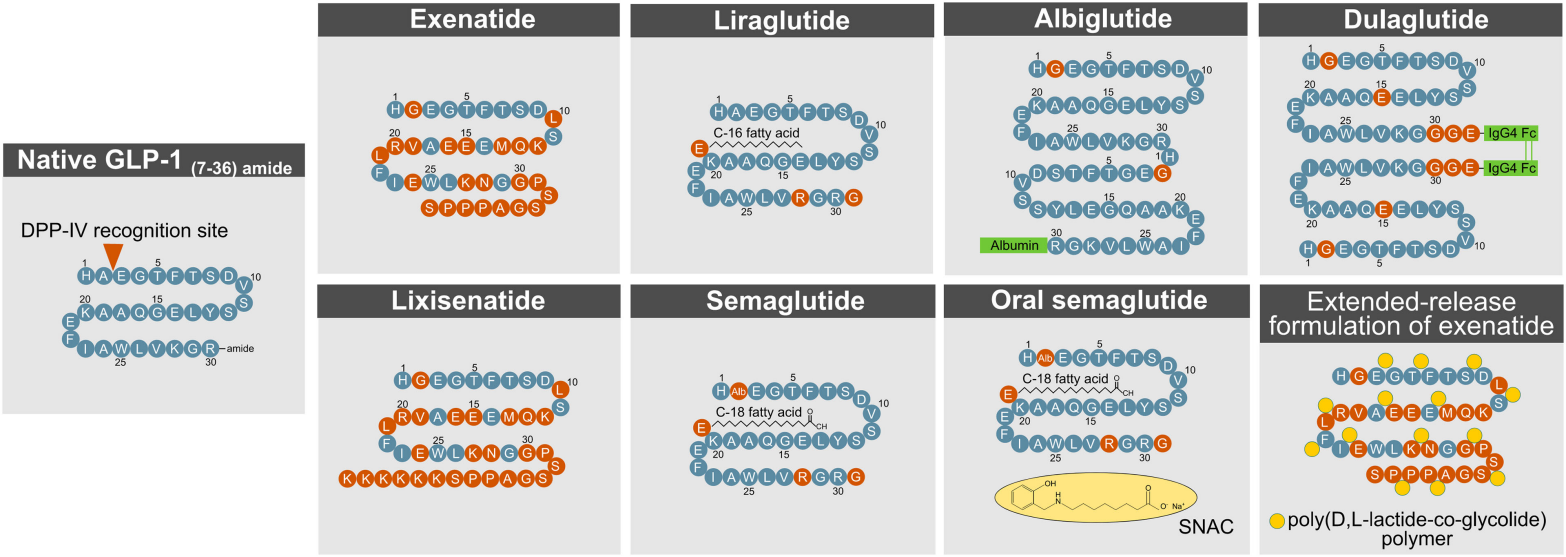
Methods to enhance GLP- 1R action. DPP- 4, dipeptidylpeptidase-4; GLP- 1, glucagon-like peptide-1.

The short action profile of native GLP-1 imposes a major challenge towards its successful clinical utilization. To overcome this limitation, various strategies have been applied to extend the half-life of GLP-1 and to accelerate its *in vivo* action and potency (**Figure 4**). The efficacy of a drug in a biological system is influenced by factors that include the stability of the active molecule and its rate of diffusion into and elimination from circulation. Methods to improve drug efficacy included structural and chemical modifications targeted to increase molecular stability and activity, to improve biodistribution/bioavailability, and to delay renal elimination (89).

#### 2.3.1 Protection From DPP-IV Cleavage

A commonly used procedure to improve the half-life of active GLP-1 is a modification at the second N-terminal amino acid position (Ala8) to protect from degradation by DPP-4. Such structural modification has been applied to exenatide, lixisenatide, semaglutide, dulaglutide, and albiglutide.

Exenatide and lixisenatide are synthetic peptides that fully resemble the sequence of exendin-4, a GLP-1 paralog that is naturally found in the saliva of the gila monster (*heloderma suspectum*). Both exenatide and lixisenatide contain the full sequence of exendin-4, but lixisenatide is extended on the C-terminus to possess six additional lysine residues (20). Exendin-4 has a glycine at the second N-terminal position (Gly8), which prevents the molecule from being fully recognized by DPP-4. Relative to native GLP-1, exendin-4 (and thus exenatide and lixisenatide) is further differentiated with amino acid substitutions in the middle segment of the sequence, which render the drug less susceptible to degradation by NEP 24.11 (62). The C-terminus of exendin-4 relative to native GLP-1 is extended by nine amino acids, which enhances its secondary structure and improves chemical stability (90). Despite being protected from DPP-4 cleavage, exendin-4 still undergoes rapid renal elimination, which in humans leads to a half-life of ~2.5 h (exenatide) and ~3–4 h (lixisenatide), respectively (29). Consistent with this notion, plasma clearance of exendin-4 is reduced 4.4-fold in nephrectomized rats (91) and 3.4-fold in patients with end-stage renal disease (92). With only 53% sequence homology to native GLP-1, exendin-4 has the limitation that ~40% of patients treated with exenatide (93–95), and ~60% of patients treated with lixisenatide (96), develop antibodies against the molecule, but this does not seem to negatively affect glucose handling or the prevalence of adverse effects. While exenatide, lixisenatide, dulaglutide, and albiglutide possess a glycine at their second N-terminal amino acid position, semaglutide bears an aminoisobutyric acid (AIB) at this residue to protect from DPP-4 inactivation.

#### 2.3.2 Covalent Binding to Albumin

Albiglutide (GlaxoSmithKline, London, UK) is a sixty amino acid tandem of two DPP-4 protected GLP-1 molecules that are covalently fused to human albumin (20). The chemical conjugation to albumin enhances GLP-1’s proteolytic stability and simultaneously delays renal elimination, which manifests as a half-life of ~120 h in humans (97). The long half-life of albumin, and potentially that of albumin-fused drugs, is derived from albumin’s ability to escape intracellular degradation by binding to the neonatal Fc receptor (FcRn), in a pH-dependent fashion (98–100). Upon endocytosis by the endothelial cells, albumin binds to the Fc receptor (FcRn) during endosomal acidification, which sorts the intracellular trafficking of the albumin away from degradative lysosomes and back to the plasma membrane where it can once again reenter the general circulation (98–100). Underlining the relevance of this intracellular mechanism, circulating levels of albumin are decreased by 40–50% in FcRn-deficient mice (99, 100). Furthermore, as the molecular size of a drug greatly affects its renal elimination (89, 101), larger molecules exhibit an inherently greater circulatory half-life due to physical hindrance in glomerular filtration (89). Thus, the fusion and binding of GLP-1 to albumin, or the linkage of several GLP-1 molecules with an Fc fragment, naturally promote a delay in renal clearance due to the increased size of the molecule.

#### 2.3.3 Acylation With a Fatty Acid

The strategic fusion of GLP-1 to a fatty acid has been applied for liraglutide (Novo Nordisk, Denmark) and semaglutide (Novo Nordisk, Denmark). Liraglutide is linked to palmitic acid (C16:0) *via* a gamma glutamic acid spacer at the lysine residue at position 26 (102). The fatty acid promotes formation of a self-associated multimolecular complex, which protracts the diffusion of the molecule from the site of injection. A delay in renal clearance of the drug is achieved by the reversible non-covalent binding of the fatty acid moiety to human serum albumin (HSA). Although liraglutide carries the native GLP-1 alanine residue at the second N-terminal position (Ala8), the fatty acid-mediated albumin binding promotes self-oligomerization and thereby improves the proteolytic stability of the molecule to further decrease the drug’s susceptibility to DPP-4 cleavage. This chemical modification manifests in a half-life of ~12 h in humans (103). Semaglutide is structurally identical to liraglutide with the exception that the Ala8 residue is substituted with AIB to further protect from DPP-IV recognition, and that the palmitic acid (C16:0) is exchanged with a dicarboxylic-stearic acid (C18:0). Impressively, these modifications manifest a half-life of ~160 h upon subcutaneous injection in humans (104). Of note, both liraglutide and semaglutide show great sequence homology (>95%) to native GLP-1, which decreases the likelihood of immunogenicity.

#### 2.3.4 Pegylation

Polyethylene glycol (PEG) is a synthetic water-soluble inert polymer with the potential to enhance a drug’s half-life by slowing down its rate of renal clearance. Pegylation of a drug can also enhance its aqueous solubility, protect against *in vivo* proteolysis, and enhance toxicological safety and potentially even its non-immunogenic resilience (105). The linkage between the PEG and a conjugated molecule/peptide can either be stable or degradable, with the latter often being used for prodrugs. Overall, pegylation does not seem to affect the folding and stability of the conjugated protein as assessed by circular dichroism, ultraviolet absorption, or NMR spectroscopy (106–110). This observation seems particularly important since misfolded proteins might lose *in vivo* potency and be subject to enhanced proteolysis and potential immunogenicity. Various FDA approved pegylated proteins are currently in therapeutic use. The first FDA approved pegylated drug was Adagen^®^ (Enzon Pharmaceuticals, new Jersey, USA) which was approved by the FDA in 1990 for adenosine deaminase deficiency associated with severe combined immunodeficiency disease (SCID). The PEG modification can, in rare cases, lead to vacuolation or the generation of antibodies against the PEG. As it pertains to drugs to control body weight and/or glycemia, preclinically tested pegylated anorectics include leptin (111), FGF21 (112–117), GIP (118, 119), GLP-1R agonists (120–122), NPY2 receptor agonists (123) and unimolecular dual-agonists targeting the receptors for GLP-1 and glucagon (124, 125) or GLP-1 and GIP (126).

#### 2.3.5 Fc Fusion

Linkage of two GLP-1 molecules *via* the Fc fragment of a monoclonal antibody (IgG4) has been applied for dulaglutide. The sixty amino acid molecule comprises two Gly8-modified DPP-4 protected GLP-1 molecules in which the C-termini are fused at a Gly36 residue to IgG4 Fc fragments. Notably, while dulaglutide carries a glycine at position 8 to protect from DPP-4 cleavage, Fc fusion to native GLP-1 is sufficient to reduce DPP-4 degradation by 4-5-fold relative to native GLP-1 (127). The Arg36 residue of native GLP-1 was substituted with Gly36 to serve as an anchor for the IgG4 Fc fragments and to decrease the possibility of T-cell epitope interaction (128). Furthermore, the native GLP-1 Gly22 residue is exchanged with glutamic acid, which stabilizes the secondary structure and enhances potency (128). The rationale of extending the half-life of GLP-1 *via* fusion to an IgG4 Fc fragment is similar to its fusion to albumin. As with albumin, the Fc complex binds upon endocytosis to the Fc receptor (FcRn) in the acidic endosomal compartments with the consequence that the FcRn-bound Fc complex is recycled back to the plasma membrane and secreted back into general circulation (101, 129–131). The Fc fragment, and the two GLP-1 motifs fused *via* the Fc fragment, also further increase the size of the molecule, and thereby naturally decrease its renal elimination (89, 101).

#### 2.3.6 Sustained-Release Formulations

Bydureon^®^ (AstraZeneca, Wilmington, USA) is an extended-release (ER) formulation of exenatide. The drug is self-applied on a weekly basis independent of meal patterns. The extended release is achieved through incorporation of exenatide (exendin-4) into 0.06 mm-diameter biodegradable microspheres, which comprise a 50:50 poly(D,L-lactide-co-glycolide) (PLG) polymer along with sucrose (132). Exenatide ER contains encapsulated exenatide at a concentration of 5 mg per 100 mg of microspheres (132). In the human body, the PLG polymers slowly degrade through the non-catalyzed hydrolysis of the ester linkages into lactic acid and glycolic acid, which are finally eliminated as carbon dioxide and water (132).

During the protracted degradation of PLG, exendin-4 is sustainably released into the general circulation for a period of several (~7) weeks, yielding therapeutic levels after two weeks and a steady state after 6–7 weeks (132). The release of exenatide into the general circulation occurs in three stages. During the initial phase, the freshly injected microspheres hydrate and immediately release loose and cell-surface-bound exenatide (< 1%) into the circulation (132). In the second phase, the polymer hydrolyzes into smaller fragments and, upon reaching a size of ~20 kDa, promotes the constant diffusion of exenatide into the circulation. In the erosion phase, the PLG polymers fully hydrolyze and eventually release all remaining exenatide (132). Reflecting this 3-phase diffusion, a first peak of exenatide release (1–2% of the total area under the plasma concentration curve) is observed in the first 48 h after the injection, followed by two peaks after approximately two and seven weeks, respectively (132).

The prolonged rise to achieve steady state plasma concentrations seems to have beneficial effects on tolerability since the frequency of nausea and vomiting is reduced in patients treated with exenatide ER relative to treatment with exenatide BID (80, 133). Exenatide ER is also superior over exenatide BID in preventing episodes of hyperglycemia, as indicated by a greater decrease in HbA1c (80). Weight loss, however, does not seem to be overtly different between patients treated with exenatide ER and exenatide BID (80). The continuous steady-state plasma concentration of exenatide (which is structurally identical in both formulations) nonetheless seems to be associated with a greater immunogenic liability for exenatide ER. In line with this notion, anti-exenatide antibodies are detected in ~70% of patients treated with exenatide ER relative to >40% of patients treated with exenatide BID (80).

#### 2.3.7 Oral Semaglutide

A general limitation to oral administration of GLP-1 mimetics is poor absorption *via* the GI-tract and quick degradation by proteolytic enzymes and the acidic environment of the stomach. For this reason, peptide GLP-1R agonists are not available as oral preparations and rather have to be subcutaneously self-injected by the patient. Difficulties and discomfort with self-injected medication is a factor that negatively affects patient compliance and quality of life (134–136). A milestone in GLP-1-based pharmacotherapies is the recent development of an oral available formulation of semaglutide (Rybelsus^®^, Novo Nordisk, Copenhagen, Denmark). In Rybelsus^®^, semaglutide is co-formulated with sodium N-[8-(2-hydroxybenzoyl)aminocaprylate] (SNAC), which shields the molecule from enzymatic and acidic degradation and accelerates its site-directed release and absorption in the stomach (137). As demonstrated in humans, the SNAC-linked semaglutide tablet undergoes surface erosion in the stomach with the result that the non-covalent linkage between semaglutide and SNAC dissolves and releases free semaglutide into the circulation (137). Another possibility to orally engage GLP-1R activity is through administration of smaller molecule mimetics. Two conventional small molecule agonist with agonism at GLP-1R have recently been disclosed and are in clinical evaluation for the oral treatment of diabetes (138–140).

### 2.4 Recent Clinical Advances of GLP-1R Agonists for the Treatment of Obesity and Diabetes

Liraglutide 3 mg (Saxenda^®^, Novo Nordisk, Copenhagen, Denmark) was approved in 2014 for the treatment of obesity in adults and in 2020 for the treatment of obesity in children aged 12 and older (**Figure 3**) (141). Between 50 and 70% of the patients treated with liraglutide 3mg achieve a body weight reduction > 5% while between 6 and 35% achieve weight loss > 10% (142). Of appreciable note, in contrast to a vast majority of previously employed anti-obesity medications (13, 20), GLP-1R agonism improves cardiovascular (CV) health in patients with T2D (48, 143). Consistent with this, a recent meta-analysis assessing CV outcome of different GLP-1R agonists across eight CV outcome trials comprising 60,080 patients with T2D. It demonstrated that GLP-1R agonists reduce the risk of a major adverse cardiovascular event (MACE) by 14%, death by 13%, non-fatal stroke by 12%, and broad composite kidney outcome by 17% (144).

In June 2021, the FDA approved semaglutide 2.4 mg (Wegovy^®^) in adjunct to lifestyle programs for the treatment of obesity in adults (145). In non-diabetic individuals overweight or obese, 68 weeks of treatment with semaglutide (2.4 mg QW), decreased body weight by 14.9% relative to 2.4% in placebo treated controls (28). Impressively, 86.4% of patients treated with semaglutide showed weight loss >5% (*vs.* 31.5% in placebo controls), while 69.1% (*vs.* 12.0% in placebo controls) lost >10%, 50.5% (*vs.* 4.9% in placebo controls) >15% and 32% (*vs.* 1.7% in placebo controls) >20% (28). Similar to the observation in patients with T2D (48, 143), participants with obesity receiving semaglutide 2.4 mg had greater improvement of markers indicative of cardiometabolic health (28). Like with other GLP-1R agonists, adverse effects of semaglutide are predominantly of gastrointestinal nature, with the most common being nausea, diarrhea, vomiting, and constipation (28). Notably, while a series of clinical studies confirms the ability of semaglutide 2.4 mg to lower body weight >10% in most patients, the magnitude of weight loss is considerably lower in diabetic relative to non-diabetic patients with obesity (28, 146–148).

## 3 Glucose-Dependent Insulinotropic Polypeptide (GIP)

### 3.1 The Physiology of GIP

GIP is derived from posttranslational cleavage of proGIP, a 153 amino acid preprohormone that is expressed in the enteroendocrine K-cells of the upper intestine, the pancreatic α-cells and potentially the CNS (149). The majority of circulating GIP refers to GIP(1-42), which gets cleaved from proGIP in the intestine by the action of PC1/3, but also a shorter form, GIP(1-30NH_2_), is produced in the intestine and the pancreatic α-cells by cleavage of proGIP by PC2 (149). Both forms are of equal insulinotropic potency in mice (150) and both carry an alanine at their second N-terminal residue, which render the molecules susceptible for DPP-4 degradation (151). Similar to GLP-1, GIP undergoes rapid renal elimination and consistent with this, intact GIP is quickly cleared from the circulation with a half-life of four minutes and without major difference between healthy subjects and individuals with T2D (151). Primarily secreted in response to the ingestion of fat, GIP promotes its biological action through binding to the GIP receptor (GIPR), a class B GPCR that, similar to GLP-1R and belonging to the glucagon receptor family (149). In mice, *Gipr* is ubiquitously expressed in the endocrine pancreas with comparable mRNA quantities in the α- β- and δ-cells (152). GIPR is further expressed in adipocytes (153), myeloid cells (154), the endothelium of the heart and blood vessels, the pituitary and the inner layers of the adrenal cortex (155). In the brain, *Gipr* is found in the cerebral cortex, hippocampus, olfactory bulb as well as in the hypothalamus and the hindbrain (155–157).

### 3.2 GIP Regulation of Lipid Metabolism

GIP is best known for its ability to act on the pancreatic islets where depending on blood glucose concentration it stimulates the secretion of insulin or glucagon (**Figure 2B**) (158). The insulinotropic effect of GIP is diminished in patients with type-2 diabetes (159) but is restored upon near normalization of glycemia upon 4-week administration of insulin (160).

Beyond its glycemic effects, GIP decreases bone resorption (161), has neuroprotective effects in animal models of Alzheimer’s disease (162), and regulates lipid metabolism (163). As comprehensively reviewed previously (149, 163–165) GIP regulation of energy and lipid metabolism is partially conflicting, since activation and inhibition of GIPR signaling can both decrease body weight and fat mass in rodents (149, 164).

As demonstrated *in vitro* in cultured adipocytes (166, 167) and *in vivo* in Zucker rats (167), GIP enhances the activity of lipoprotein lipase (LPL), which promotes adipose tissue lipid disposal by enhancing hydrolytic cleavage of circulating triglycerides (TAG) into fatty acids (FA) and monoacylglycerol, which gets re-esterified and stored in adipose tissue. Consistent with this, GIP promotes clearance of circulating TAGs in dogs (168) and enhances adipose tissue TAG accumulation in rats (169) and humans (170). Under conditions of hyperglycemia and hyperinsulinemia, GIP increases adipose tissue blood flow and TAG clearance in healthy lean subjects (171). It further enhances FA synthesis in adipose tissue explants (172) and potentiates insulin-stimulated uptake of FA into the adipocytes (173). Collectively, GIP can act on the adipose tissue to increase adipose tissue blood flow, to enhance LPL-induced TAG clearance from the circulation and to stimulate adipocyte lipid storage. Recently, it was hypothesized that GIP may also facilitate healthy white adipose tissue expansion and thereby protect from adipocyte lipid spill-over and ectopic accumulation of lipids (163). This hypothesis is anchored on the observations that GIP is a target of PPARγ, a master regulator of adipogenesis (174), the expression of GIP increases during adipocyte differentiation (175, 176), and that knockdown of GIPR impairs adipocyte development (175).

### 3.3 Regulation of Energy Metabolism by GIPR Agonism and Antagonism

Consistent with the proposed role of GIP in lipid storage, mice with global loss of *Gipr* are lean and exhibit decreased body weight gain when fed a high-fat diet (HFD) (177). Body weight is also decreased in *Gipr* deficient ob/ob mice relative to ob/ob controls (177) and is decreased in mice with deletion of *Gipr* in the adipose tissue (178) and the CNS (179). However, the body weight difference between wt mice and CNS- or adipose-specific *Gipr* ko mice is only mild in comparison to the global loss of *Gipr* (177–179), indicating that lack of GIPR signaling in these tissues does not fully explain the phenotype of the global *Gipr* ko mice. No difference in body weight is seen between wt mice and mice that lack *Gipr* in the brown adipose tissue (BAT) (180) or the pancreatic β-cells (181).

The observation that global loss of *Gipr* protects from diet-induced obesity (177) and that GIP can promote adipocyte lipid storage (163) has inspired the development of GIPR antagonists for the treatment of obesity (182). Among the most prominent GIPR antagonists are natural (183) and biochemically modified (fatty-acylated) (184) forms of N-terminally truncated GIP as well as GIPR neutralizing antibodies (181, 185, 186). When administered peripherally, particularly antibody-based GIPR antagonists demonstrate some potential to prevent the development of HFD-induced obesity in rodents (181, 186), but GIPR antagonists show only modest, if any ability to decrease body weight once obesity is already established (181, 183–186). However, one study demonstrated that central administration of an antibody-based GIPR antagonist remarkably decreased body weight in DIO mice, potentially through mechanisms that include restoration of leptin sensitivity (185). Enhanced leptin action can however not be the sole mechanism by which centrally applied GIPR antagonists decrease body weight, since loss of GIPR in leptin deficient ob/ob mice still decreases body weight relative to ob/ob controls (177).

While GIPR antagonism has only little effects to decrease body weight in DIO mice (181, 183–186), its combination with GLP-1R agonism decreases body weight beyond what is possible with either monotherapy alone (181). Interestingly, this observation is surprisingly similar to long-acting (acylated) GIPR agonists when co-injected with fatty-acyl GLP-1 (126). When given at a dose of 3 nmol/kg/day, fatty-acyl GIP fails to affect body weight in DIO mice, but when given as an adjunct to fatty-acyl GLP-1 (3 nmol/kg/day), weight loss in the co-therapy is synergistically greater than treatment with GIP or GLP-1 alone (126). The mechanism of how GIPR and GLP-1R agonism synergizes to enhance body weight loss are not known, but a series of experimental results indicates that GIP acts on the GIP receptor in the brain to decrease body weight *via* inhibition of food intake (156, 179, 187). Consistent with this, GIPR is expressed in the hypothalamus (156) and the hindbrain (157, 188) and DREADD-mediated activation of GIPR neurons/cells in the hypothalamus decreases food intake in rodents (156). Central and peripheral administration of a long-acting (fatty-acylated) GIP increases cFOS neuronal activation in key feeding areas of the hypothalamus (179) and several long-acting GIPR agonists have been shown to decreases body weight and food intake in DIO mice without affecting energy expenditure (179, 184). How pharmacological activation and inhibition of GIPR both improve energy and lipid metabolism is subject of intense scientific investigation. Relevant hypotheses include that GIPR agonists may desensitize GIP receptor signaling (189), or that GIPR agonists and antagonists improve metabolism through independent mechanisms (187). Arguing against a role of GIPR agonists as functional antagonists is the observation that single central administration of acylated GIP is sufficient to rapidly lower food intake within hours after its administration (179) and that even DREADD-mediated (non-GIPR ligand-induced) activation of GIPR neurons decreases food intake in mice (156). Also, expression of GIPR is not decreased upon chronic treatment of DIO mice with acyl-GIP in either the hypothalamus or the adipose tissue (179) and dual-agonists targeting the receptors for GLP-1 and GIP do not show enhanced internalization of the GIP receptor (42, 190).

The ability of GIPR agonists to decrease body weight and food intake vanishes in global *Gipr* ko mice but is preserved in GLP-1R ko mice (179, 184), indicating that GIPR agonists lower body weight and food intake independent of GLP-1R signaling *via* the GIP receptor. When administered centrally (icv), fatty-acyl GIP decreases body weight and food intake in HFD-fed wildtype mice but not in mice with CNS deletion of *Gipr* (179). When administered peripherally, fatty-acyl GIP fails to affect food intake in CNS *Gipr* ko mice but shows partially preserved ability to decrease body weight (179). These data indicate that fatty-acyl GIP acts in the brain to decrease body weight *via* inhibition of food intake but also decreases body weight independent of GIPR in the CNS (179). Although GIPR agonists do not require GLP-1R to decrease body weight (179, 184), GIPR agonism was recently shown to attenuate the emetic effects of GLP-1R agonism in mice, rats and musk shrews (188). Given the role of the caudal hindbrain in emetic/aversive behavior (191) and in mediating GLP-1 effects on food intake (56, 192, 193), these data collectively suggest that GIP potentially acts on the hindbrain-hypothalamus axis to decrease food intake and to improve tolerability of GLP-1R agonism.

### 3.4 GIPR/GLP-1R Dual-Agonists

The observation that weight loss in DIO mice is enhanced by co-therapy with GIPR and GLP-1R agonists (126) has resulted in the development of unimolecular GLP-1R/GIPR dual-agonist peptides (82, 126). The first of such dual-agonist, MAR709, shows nearly balanced activity at both target receptors and improves body weight and glycemia with greater potency relative to pharmacokinetically-matched GLP-1 in rodents with diet- and genetically induced obesity (126). In a 12-wk phase II study, MAR709 (a.k.a. NNC0090-2746) reduced body weight and blood glucose in patients with T2D, but the drug candidate at the single tested dose was not superior to dose titrated treatment with liraglutide (83). In 2020, Novo Nordisk announced discontinuation of MAR709 due to success of semaglutide 2.4 mg in clinical trials and in favor of other potentially more effective drugs in their clinical pipeline (13).

Another GIPR/GLP-1R co-agonist, Tirzepatide (a.k.a. LY3298176) has been developed by Eli Lilly (82). The molecule is based on the human GIP sequence, in which GLP-1R residues were introduced to yield a fivefold greater potency at the human GIPR relative to GLP-1R (82). Fatty-acylation of the lysine 20 residue with a C20 diacid allows covalent binding to albumin, which results in a half-life of ~160 hrs in humans (82). When given at equimolar concentrations, tirzepatide shows greater weight loss but equal improvement in glucose metabolism relative to treatment with semaglutide in DIO mice (82). Interestingly, when given at a daily dose of 10 nmol/kg, tirzepatide improves glucose metabolism but fails to affect body weight in GLP-1R ko mice (194). These data are seemingly in contrast to previous reports showing preserved ability of long-acting GIPR agonists to decrease body weight in GLP-1R ko mice (179, 184). Importantly, human GIP is only a weak and partial agonist at the mouse relative to the human GIP receptor (195), and all currently available data on the pharmacokinetics of tirzepatide are based on human GIPR (82). It warrants clarification whether tirzepatide shows preserved potency at the mouse GIPR.

In recent phase III clinical trials, tirzepatide showed profound ability to improve body weight and glycemia in patients with obesity and T2D. Depending on the dose (5, 10, or 15 mg QW), 40-52 weeks of treatment with tirzepatide, decreased HbA1c between -1.87 and – 2.59%, with 81 – 97% of patients achieving a HbA1c <7%, and 23 – 62% of patients achieving HbA1c <5.7% (24–27, 196). Serum levels of fasting glucose decreased between 43.6 – 67.9 mg/dl while body weight decreased relative baseline between 6.2 – 12.9% (24–27, 196). After 40 weeks of treatment, 47 – 57% of patients treated with tirzepatide 15mg QW decreased body weight >10%, relative to 1% in patients treated with placebo, while 27 – 32% of patients decreased body weight >15% (0% in placebo controls) (25, 27, 196). Treatment with tirzepatide was at all tested doses (5, 10, 15 mg QW) superior to treatment with semaglutide 1mg QW to decrease HbA1c, fasting levels of blood glucose and body weight (25). It warrants to be determined how tirzepatide compares to the recently approved semaglutide 2.4 mg. Interestingly, while tirzepatide shows comparable ability to decrease body weight in obese type-2 diabetic vs non-diabetic individuals (24–27), Semaglutide 2.4 mg is considerably less efficacious in obese diabetic relative to obese non-diabetic individuals (28, 146–148).

## 4 Outlook

The results of completed tirzepatide trials are quite exciting. However, some effort is still required to ensure such therapeutic advance will be applicable to all populations for whom it is intended. Racial and ethnic minorities carry a disproportionate burden of obesity and T2D in the general population, but their enrolment in the completed tirzepatide trials was lower than expected, which raises concerns regarding the generalizability of these trials. Female participants were reasonably represented in the completed trials, but no assessment of biological sex differences in drug efficacy and safety profiles was made (24–27, 196). T2D is increasingly diagnosed in children, adolescents, and young adults; there is a need for more efficacious, reliable therapies approved for younger patients. The heterogeneous and multifactorial etiology of T2D and obesity combined with the physiologic complexity of human metabolic system contributes to wide variations in clinical phenotypes and individual response to treatment. Adequate demographic representation across races and ethnic groups, geographic areas, sexes and genders, and age groups may lead to more robust and complete data that broaden the understanding of inter-individual response variability, which may in turn help expand the population of patients responsive to new medications such as tirzepatide.

It is well recognized that T2D and obesity treatments may be best suited for precision medicine approaches rather than a “one size fits all” paradigm. While considerable effort continues to be invested into developing more pharmacotherapies for T2D and diabetes, it is of paramount importance that use of the currently available medications is optimized—that is, to provide the right treatment to the right patient, at the right time. The completed trials of tirzepatide demonstrate meaningful improvement of glycemic and weight control with this drug. However, as with any medical therapy, there are large inter-individual differences in response among participants, including the possibility of less than 5% weight loss to 20% or greater weight loss (24–27, 196), suggesting that individualization and precision treatment might be necessary to achieve greater disease modification. Thus, the challenge for scientists and physicians is to identify and validate biomarkers for drug selection. In future trials, accurate and timely detection of individual response to investigational drugs and reporting individual response data will be helpful in differentiating treatment responders from non-responders. The overall goal is to utilize specific genetic, clinical, and biochemical characteristics of individual patients to tailor treatment to achieve optimal outcomes. Moreover, from a clinical perspective, it will be equally important to decipher mechanisms by which GIP acts on the brain to decrease body weight and to determine which brain areas are involved. Much further work is also needed to establish how GIPR antagonism decreases body weight (centrally or peripherally mediated) and how GIPR and GLP-1R agonism synergizes to decrease body weight.

## Author Contributions

QT and SA co-wrote sections and edited the manuscript. CO made the figures and edited the manuscript. RW edited the manuscript. RD edited and revised the manuscript and co-wrote sections. TM conceptualized and wrote the manuscript. AH co-conceptualized the manuscript, co-wrote sections, and edited the manuscript. All authors contributed to the article and approved the submitted version.

## Funding

QT received research funding from the International Helmholtz Research School for Diabetes and the Alberta Diabetes Institute. SA received research funding from the Helmholtz Association (Helmholtz Diabetes School). TM received research funding from the German Research Foundation (TRR152 and TRR296). AH received research funding from the Canadian Institutes of Health Research and Weston Family Foundation (Pro00069477 and Pro00100067).

## Conflict of Interest

AH is an investigator on clinical trials for Rhythm Pharmaceuticals, Inc., and Levo Therapeutics, has received grant funding from the W. Garfield Weston Foundation, and has served as a speaker for Rhythm Pharmaceuticals, Inc. TM receives research funding by Novo Nordisk, but these funds are unrelated the here described work. TM further received speaking fees from Eli Lilly, Novo Nordisk, Mercodia, AstraZeneca, Berlin Chemie, and Sanofi Aventis. RD is a co-inventor on intellectual property owned by Indiana University and licensed to Novo Nordisk. He was recently employed by Novo Nordisk and previously Lilly Research Laboratories.

The remaining authors declare that the research was conducted in the absence of any commercial or financial relationships that could be construed as a potential conflict of interest.

The handling editor declared a shared affiliation with several of the authors SA and TM.

## Publisher’s Note

All claims expressed in this article are solely those of the authors and do not necessarily represent those of their affiliated organizations, or those of the publisher, the editors and the reviewers. Any product that may be evaluated in this article, or claim that may be made by its manufacturer, is not guaranteed or endorsed by the publisher.
